# Species-Specific Responses of Kiwifruit Seed Germination to Climate Change Using Classifier Modeling

**DOI:** 10.3390/plants14172665

**Published:** 2025-08-26

**Authors:** Tung-Yu Hsieh, Feng Li, Shih-Li Huang, Ching-Te Chien

**Affiliations:** 1 School of Food and Bioengineering, Fujian Polytechnic Normal University, Fuqing 350300, China; a0920169868@gmail.com; 2 Fujian Universities and Colleges Engineering Research Center of Modern Facility Agriculture, Fujian Polytechnic Normal University, Fuqing 350300, China; 18850659211@139.com; 3 Lanxi Agricultural Technology Co., Ltd., Fuqing 350300, China; 4 Wuyou Ecological Agriculture Co., Ltd., Fuqing 350300, China; 5 Cai Die Biological Technology Co., Ltd., Yongtai 350700, China; 6 Fujian Tianyuan Shiguang Leisure Agriculture Co., Ltd., Fuqing 350300, China; 7 Fujian Rongtai Yidu Agricultural Technology Co., Ltd., Fuqing 350300, China; 8 Department of Forestry, National Chung Hsing University, Taichung City 40227, Taiwan

**Keywords:** climate adaptation, seed dormancy, phenological modeling, ecological resilience, classifier modeling, kiwifruit conservation, *Actinidia* species, global warming impact, bioclimatic stress responses

## Abstract

Climate change is reshaping plant reproductive processes, particularly at the vulnerable seed germination stage. This study examines the germination responses of four *Actinidia* species (*A. rufa, A. latifolia, A. deliciosa,* and *A. setosa*) under controlled experimental conditions, integrating empirical germination data with classifier modeling to predict species-specific responses under future climate scenarios. Unlike traditional species distribution models (SDMs), our classifier approach incorporates physiological dormancy mechanisms and key environmental cues such as chilling requirements, temperature fluctuations, and drought stress. Results reveal significant interspecific differences: *A. rufa* exhibited strong ecological plasticity, maintaining stable germination under warming and drought, while *A. deliciosa* displayed extreme sensitivity to warming, with germination dropping below 25% due to its strict chilling requirement. *A. latifolia* showed latitude-dependent vulnerability, with southern populations experiencing reduced germination under warming conditions, and *A. setosa* demonstrated complex dormancy patterns with higher germination at high elevations. The predictive accuracy of our models was validated against long-term field data, underscoring their robustness in forecasting climate-induced germination shifts. These findings highlight the need for targeted breeding programs to develop *A. deliciosa* cultivars with reduced chilling requirements and suggest *A. rufa* as a strong candidate for ecological restoration under future warming scenarios. By refining climate impact assessments through physiological modeling, this study provides valuable insights for kiwifruit conservation, agricultural adaptation, and broader plant-climate interactions under global warming.

## 1. Introduction

Anthropogenic climate change is fundamentally reshaping global ecosystems, driving shifts in species distributions, altering phenological patterns, and disrupting ecological dynamics [1,2]. Since the onset of the Industrial Revolution, rising atmospheric greenhouse gas concentrations have led to significant increases in temperature, alterations in precipitation regimes, and more frequent extreme weather events, imposing complex stressors on plant communities [3,4,5]. Among these challenges, changes in seasonal temperature regimes pose severe threats to plant reproductive success, particularly during the critical stages of seed dormancy release and germination [6,7]. Understanding how plant species respond to climate-driven stressors is essential for predicting future distribution patterns and developing adaptive strategies for conservation and agriculture [8,9].

Seed germination is one of the most climate-sensitive phases in a plant’s life cycle, directly influencing population persistence, recruitment success, and ecosystem resilience under shifting environmental conditions [10,11,12,13]. While significant research has focused on the impact of climate change on flowering and fruiting phenology [14], relatively little attention has focused on climate-induced alterations in seed dormancy mechanisms and germination dynamics [15]. Temperature shifts may disrupt dormancy-breaking cues, alter stratification requirements, and impose physiological constraints on seed viability [16], ultimately affecting species survival and community structure [17]. Given the ecological and economic importance of seed-based regeneration, a deeper understanding of species-specific germination responses to climate variability is urgently needed.

Traditional species distribution models (SDMs) have been widely employed to predict plant responses to climate change based on ecological niche theory [18,19]. However, most SDMs rely on macroclimatic variables and often fail to incorporate species-specific physiological traits [20], such as chilling requirements, dormancy-breaking thresholds, and microhabitat preferences. This limitation can lead to overestimations or underestimations of species’ climatic tolerances and adaptive capacities [8]. Addressing this gap requires an integrative modeling approach that combines empirical germination data with statistical classification frameworks to improve the accuracy of climate impact assessments.

The genus *Actinidia* (kiwifruit) is an ecologically and commercially significant taxon, widely distributed across East and Southeast Asia [21,22]. Taiwan hosts several indigenous *Actinidia* species alongside the widely cultivated *A. deliciosa* (A. Chev.) C.F. Liang & A.R. Ferguson [23,24], making this genus an ideal model for investigating species-specific seed germination responses to climate change. These species occupy diverse altitudinal and latitudinal gradients, allowing for a comprehensive examination of how environmental factors such as winter warming, drought stress, and elevation-dependent dormancy shape germination patterns [25]. Despite their ecological importance, the effects of climate change on *Actinidia* seed germination remain poorly understood, particularly concerning chilling requirements, dormancy mechanisms, and their resilience to warming and drought conditions.

This study aims to address these knowledge gaps by systematically investigating the seed germination responses of four *Actinidia* species (*Actinidia rufa* (Siebold & Zucc.) Planch. ex Miq., *A. latifolia* (Gardner & Champ.) Merr., *A. deliciosa,* and *A. setosa* (H.L. Li) C.F. Liang & A.R. Ferguson) under controlled experimental conditions. By integrating empirical germination data with classifier modeling, we simulate germination dynamics under projected climate scenarios, focusing on temperature-dependent dormancy shifts, drought stress tolerance, and elevational and latitudinal variations in germination success. Our study presents a novel application of classifier models, providing a quantitative framework for assessing species-specific resilience and vulnerability to climate stressors.

By refining our understanding of climate-driven germination responses, this study contributes to the broader field of plant ecophysiology, climate adaptation, and conservation biology. The findings are expected to inform conservation strategies for wild *Actinidia* populations, guide breeding programs for climate-resilient kiwifruit cultivars, and enhance predictive models of species distributions under future warming scenarios. As global temperatures continue to rise, integrating physiological and ecological modeling approaches will be critical for developing adaptive management strategies to mitigate the impacts of climate change on plant reproductive success.

## 2. Materials and Methods

### 2.1. Study Species and Seed Collection

The genus *Actinidia* (*kiwifruit*) is widely distributed across East and Southeast Asia, encompassing species with diverse ecological adaptations. This study focuses on four *Actinidia* species—*A. rufa*, *A. latifolia*, *A. deliciosa*, and *A. setosa*—to investigate interspecific variation in seed dormancy and germination responses under projected climate change scenarios. These species were selected due to their broad ecological range, economic significance, and distinct physiological adaptations to environmental stressors, making them ideal models for assessing climate-induced shifts in germination dynamics.

Mature fruits were collected during the peak fruiting season (September to November) from natural forests and cultivated orchards across Taiwan. To capture the full range of environmental variability, seeds were sampled across different elevations and latitudes:

- *A. setosa*: Siyuan Yakou, Yilan County (1870 m); Cuifeng, Nantou County (2250 m)
- *A. deliciosa*: Shengguang, Heping District, Taichung City (2500 m)
- *A. rufa*: Near Qingjing Farm, Nantou County (1800 m)
- *A. latifolia*: Beidongyan Mountain, Nantou County (1600 m); Wufeng, Taichung County (200 m); Mudan Township, Pingtung County (400 m)

Fruits were stored at ambient temperature until fully ripened. Seeds were extracted by pulp removal, and non-viable seeds were discarded using the flotation method. The remaining viable seeds were air-dried at 25 °C for 48 h and subsequently stored in airtight containers at 5 °C with 33% relative humidity (RH) until further experimentation.

### 2.2. Experimental Design

To assess seed dormancy and germination responses, a controlled growth chamber experiment was conducted. Three primary environmental stressors—cold stratification, temperature fluctuation, and drought stress—were examined through a series of factorial germination trials.

#### 2.2.1. Cold Stratification Treatments

To evaluate the influence of chilling duration on dormancy release, seeds were subjected to five cold stratification periods (0, 2, 4, 8, and 12 weeks) at 5 °C in moist sphagnum moss. Following stratification, seeds were sown in Petri dishes lined with moistened filter paper and incubated at 25/15 °C (12-h photoperiod, 60–80 µmol m^−2^ s^−1^ photon irradiance) [26,27].

#### 2.2.2. Temperature and Warming Treatments

To simulate the impact of projected climate warming on winter temperatures in a subtropical region, seeds were incubated under four temperature regimes reflecting anticipated climate scenarios [28,29]:

- No warming (15/6 °C): This serves as the control group, representing the current or baseline climate conditions. It allows researchers to compare how seed germination performs under the current climate compared to warmer scenarios.
- Mild warming (20/10 °C): This level simulates a slight increase in temperature, reflecting a potential future climate scenario with minimal warming.
- Moderate warming (25/15 °C): This represents a more significant temperature increase, corresponding to a scenario with moderate climate warming.
- Extreme warming (30/20 °C): This level simulates a substantial temperature rise, indicative of a future climate with significant warming. It helps researchers understand how seeds might respond to more extreme temperature conditions.

#### 2.2.3. Drought Stress Treatments

To assess the effects of moisture limitation on germination, seeds were subjected to different dry storage durations before rehydration and germination testing [29]:

- Mild drought: 2 weeks of dry storage (RH 33%)
- Moderate drought: 4–8 weeks of dry storage
- Severe drought: ≥12 weeks of dry storage

After the designated storage period, seeds were rehydrated and incubated under the control temperature regime (25/15 °C) to assess germination recovery.

### 2.3. Germination Assessment and Data Collection

Germination was monitored weekly for 8 weeks, and a seed was recorded as germinated upon radicle emergence (>2 mm). Each treatment was replicated three to four times, with 50–100 seeds per replicate.

To minimize experimental bias:

- Germination conditions were maintained in a controlled-environment growth chamber (relative humidity: 70%; light/dark cycle: 12/12 h; photon irradiance: 60–80 µmol m^−2^ s^−1^).
- Watering and seed hydration conditions were standardized to prevent variation in moisture levels.
- Germination percentages were calculated as:

$$Germination\;percentages\left(\%\right)=\frac{number\;of\;germinated\;seeds}{total\;number\;of\;viable\;seeds}\times 100$$

### 2.4. Classifier Modeling and Model Validation

A logistic regression-based classifier model, referred to as a dichotomizer [23], was developed using R version 4.3.3 [30] to assess the probability of seed germination under different environmental conditions. Logistic regression is particularly suited for binary outcome prediction (germination: 1, non-germination: 0), allowing for robust quantification of species-specific germination responses to climate-driven stressors. Model training was iteratively performed until a 100% classification accuracy was achieved on the training dataset, at which point the model was considered fully trained.

#### 2.4.1. Model Structure and Predictors

The general form of the logistic regression classifier model is:

$$g=\frac{e^{\alpha +\beta_{i}x_{i}}}{1+e^{\alpha +\beta_{i}x_{i}}}\times 100$$

where *g* (germination) is the probability of germination, *X_i_* represents explanatory variables (e.g., temperature, chilling duration, altitude, latitude, drought duration), and *β_i_* are the estimated coefficients.

Six logistic regression models were developed to examine specific germination drivers:

1. Chilling Duration Model: Influence of cold stratification on germination success.
2. Winter Warming Model: Effect of increasing temperature on *A. setosa, A. deliciosa,* and *A. rufa* germination.
3. Altitudinal Model: Relationship between germination percentage and elevation for *A. setosa* and *A. latifolia*.
4. Latitudinal Model: Impact of latitude on *A. latifolia* germination under warming conditions.
5. Drought Model: Effect of dry storage duration on germination probability for *A. latifolia, A. deliciosa,* and *A. rufa*.
6. Combined Stress Model: Interactive effects of warming and drought on *A. deliciosa* and *A. rufa*.

#### 2.4.2. Model Validation with Field Data

To validate the models’ accuracy and ecological applicability, predicted germination responses were compared with long-term field distribution data of *Actinidia* species across Taiwan [31]. The observed species distributions under varying climatic conditions were analyzed to assess whether model predictions aligned with real-world germination success and recruitment patterns. A strong correspondence between modeled predictions and field observations would support the models’ utility for forecasting climate-induced germination shifts.

## 3. Results

### 3.1. Seed Germination Responses Across Different Species and Elevations

The germination responses of the four *Actinidia* species exhibited substantial variation across elevations and environmental conditions, as illustrated in Table 1. *A. latifolia* seeds collected from the low-elevation site in southern Taiwan (Mudan, Pingtung County, 400 m) showed a notably low germination percentage of 43.0%, whereas those from the low-elevation site in central Taiwan (Wufeng, Taichung County, 200 m) demonstrated a significantly higher germination rate of 96.3%. This stark contrast suggests that regional climatic differences or genetic adaptations may influence germination success at similar elevations. Seeds from the higher-elevation site (Beidongyan Mountain, Nantou County, 1600 m) exhibited a germination rate of 94.8%, aligning closely with the central low-elevation population.

For *A. setosa*, which primarily inhabits higher elevations, the initial germination percentages were relatively low, with 8.0% at 1870 m and 10.7% at 2250 m. Cold stratification at 5 °C moderately improved germination to 16.0% and 14.0%, respectively, indicating that *A. setosa* exhibits deep dormancy, which is only partially alleviated by chilling exposure.

*A. rufa*, collected from an elevation of 1800 m, displayed inherently high germination percentage (89.3%), with a marginal increase to 90.7% following cold stratification. These results suggest that *A. rufa* seeds do not require prolonged chilling exposure to break dormancy, indicating a high level of ecological adaptability.

In contrast, *A. deliciosa* exhibited a strong dependence on chilling for dormancy release. Seeds collected from the highest elevation (2500 m) initially showed a very low germination rate of 3.3%. However, after cold stratification, germination increased drastically to 93.3%, underscoring this species’ strong requirement for chilling accumulation before germination.

### 3.2. Impact of Low Temperature on Germination

**Figure 1:**
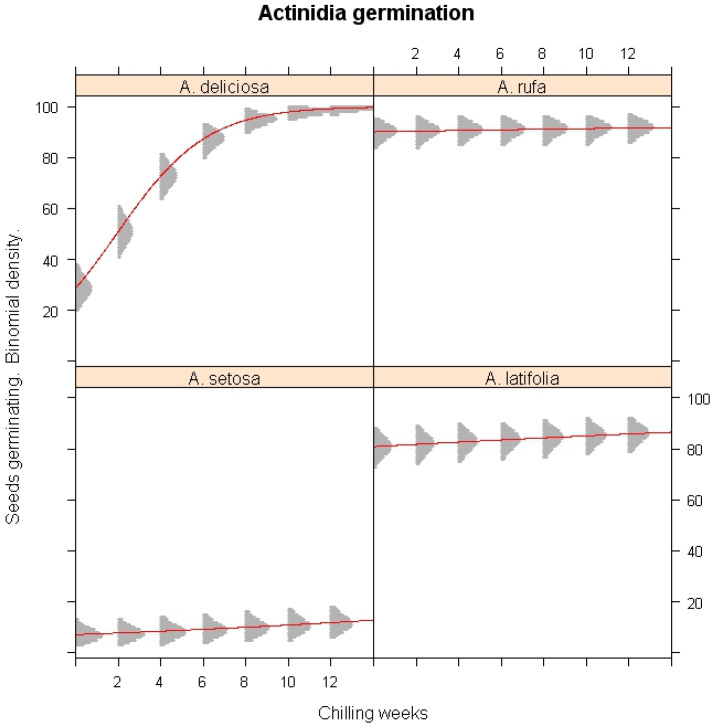
Simulations of the impact of winter chilling duration on the seed germination of *A. deliciosa*, *A. rufa*, *A. setosa*, and *A. latifolia* under different chilling weeks. The x-axis represents continuous stratification duration; the y-axis shows predicted germination probability. The red lines represent the logistic regression lines, and the gray shaded areas are binomial confidence intervals from the logistic regression model.

The effect of cold stratification on seed germination was further evaluated using classifier model analysis (Figure 1). Low-temperature exposure significantly enhanced the germination of *A. deliciosa* (*p* < 0.001), demonstrating its strict chilling requirement. In contrast, germination in *A. rufa*, *A. setosa*, and *A. latifolia* was not significantly influenced by chilling exposure (*p* > 0.05), confirming that these species do not rely on extended cold exposure for dormancy release. The simulation results revealed that eight weeks of chilling were sufficient to break dormancy in *A. deliciosa*, while *A. setosa* exhibited persistent dormancy even under prolonged chilling conditions.

### 3.3. Effects of Winter Warming on Germination

**Figure 2:**
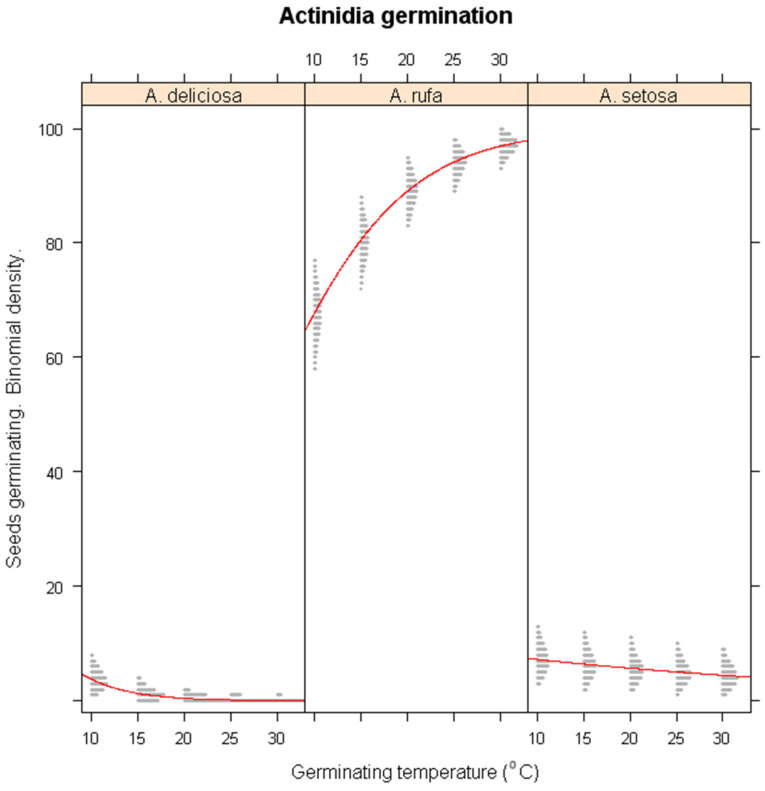
Simulations of warming winter impacts on the seed germination of *A. deliciosa*, *A. rufa* and *A. setosa* under different temperature regimes. The x-axis represents germination temperature.; the y-axis shows predicted germination probability. The red lines represent the logistic regression lines, and the gray shaded areas are binomial confidence intervals from the logistic regression model.

Under simulated winter warming conditions, significant species-specific differences in germination responses were observed (Figure 2). *A. deliciosa* showed a significant reduction in germination as temperatures increased (*p* < 0.01), emphasizing its reliance on cold exposure for germination. In contrast, *A. rufa* benefited from warmer conditions, with germination percentage increasing significantly (*p* < 0.001), suggesting that this species is adapted to milder winter conditions. While *A. setosa* exhibited a slight decline in germination under warming conditions, this effect was not statistically significant (*p* > 0.1). These results indicate that *A. deliciosa* is highly vulnerable to climate-induced warming, whereas *A. rufa* may exhibit increased germination success under future warming scenarios.

### 3.4. Altitudinal and Latitudinal Variability in Germination

**Figure 3:**
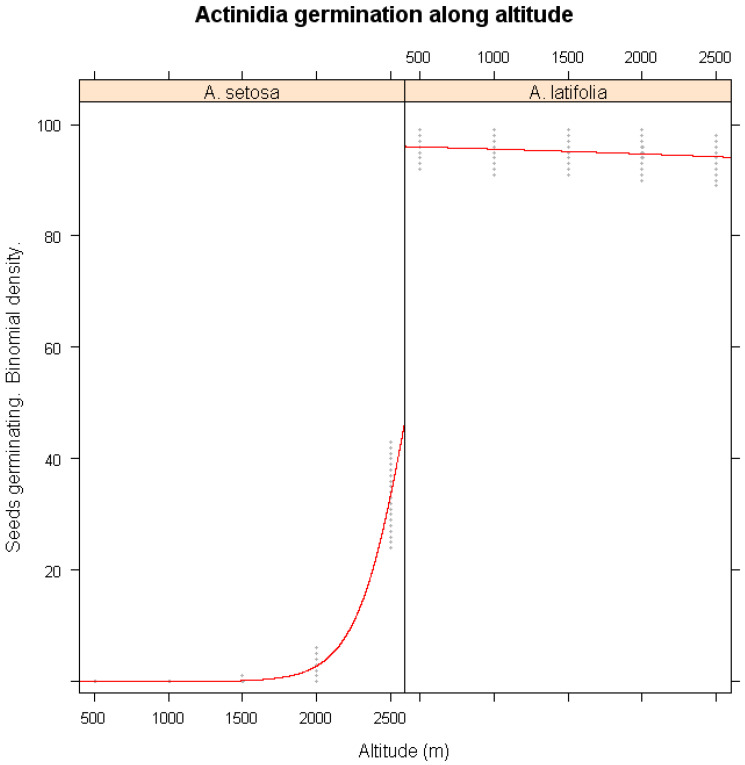
Simulations of the impact of winter warming on the seed germination of *A. setosa* and *A. latifolia* along altitude gradients of their habitats. The x-axis represents the altitude of seed provenances, and the y-axis shows predicted germination probability. The red lines represent the logistic regression lines, and the gray shaded areas are binomial confidence intervals from the logistic regression model.

The effects of altitude on germination responses were examined in Figure 3. *A. setosa* seeds from lower elevations exhibited a pronounced decline in germination, while those from higher elevations maintained relatively stable germination percentage. This suggests that populations at lower elevations may be more sensitive to climate fluctuations, possibly due to genetic differentiation or local adaptation. In contrast, *A. latifolia* did not show a significant correlation between altitude and germination success (*p* > 0.05), indicating that its germination is influenced more by other environmental factors.

**Figure 4:**
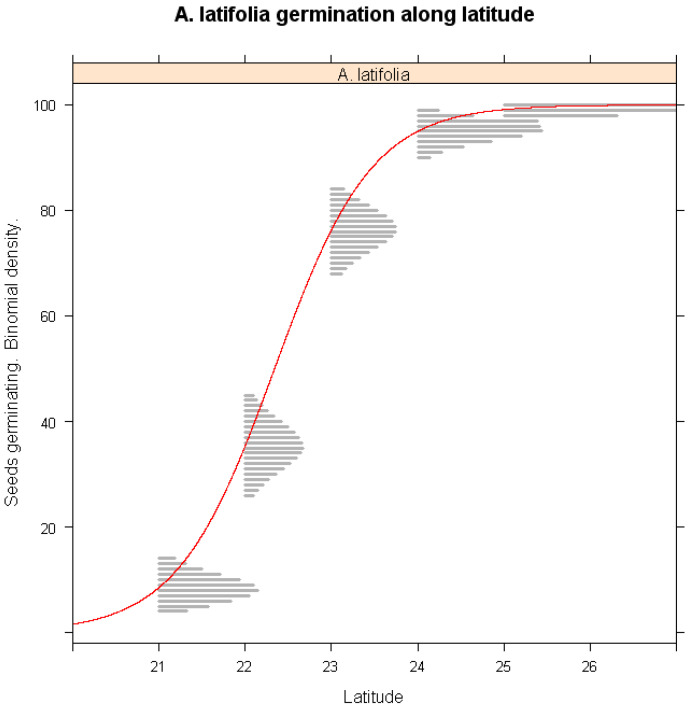
Simulations of warming winter impacts on the seed germination of *A. latifolia* along latitude gradients of its habitats. The x-axis represents the latitude of seed provenances, and the y-axis shows predicted germination probability. The red lines represent the logistic regression lines, and the gray shaded areas are binomial confidence intervals from the logistic regression model.

The influence of latitude on germination success was assessed in Figure 4. *A. latifolia* populations from southern Taiwan exhibited significantly lower germination percentage under warming conditions (*p* < 0.001), whereas populations from northern Taiwan remained largely unaffected. This suggests that southern populations are more vulnerable to climate-induced temperature increases, likely due to differences in their thermal tolerance thresholds or genetic adaptations. These findings highlight the importance of considering latitude-dependent responses in conservation and breeding strategies for *A. latifolia*.

### 3.5. Influence of Winter Drought on Germination

**Figure 5:**
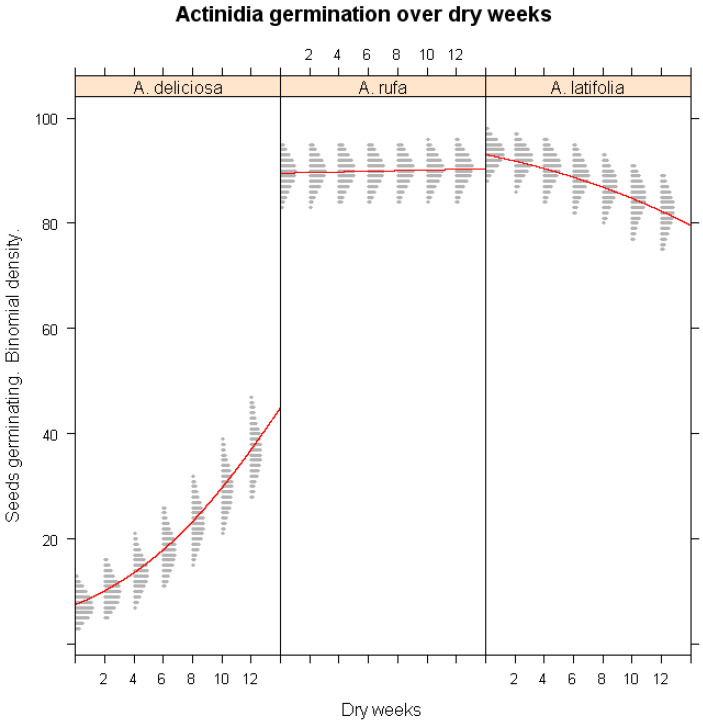
Predicted seed germination probability of three *Actinidia* species (*A. deliciosa*, *A. rufa*, and *A. latifolia*) under different durations of winter drought. The x-axis represents continuous drought duration; the y-axis shows predicted germination probability. The red lines represent the logistic regression lines, and the gray shaded areas are binomial confidence intervals from the logistic regression model.

Winter drought conditions had a notable impact on seed germination across species (Figure 5). *A. deliciosa* exhibited an unexpected slight increase in germination under prolonged drought exposure, suggesting that a portion of dormancy release in this species may be triggered by desiccation stress. Conversely, *A. latifolia* showed a sharp decline in germination with extended drought exposure (*p* < 0.001), indicating its high sensitivity to moisture deficits. The germination of *A. rufa* remained largely unaffected by drought stress, further reinforcing its classification as a highly resilient species. These findings suggest that *A. latifolia* may require targeted conservation strategies to mitigate the negative impacts of prolonged drought conditions.

### 3.6. Combined Effects of Winter Warming and Drought Stress

**Figure 6:**
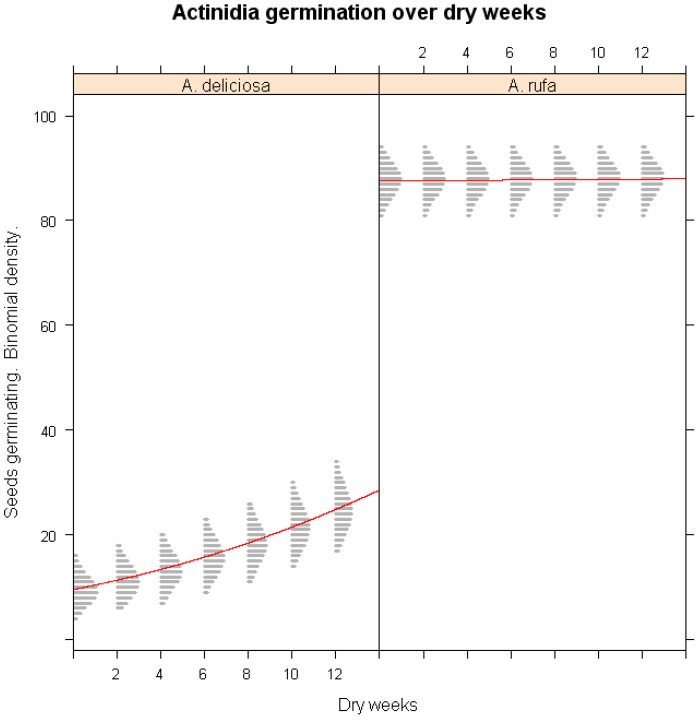
Predicted seed germination probability of *Actinidia deliciosa* and *A. rufa* under severe warm winter conditions following different drought durations. The x-axis represents continuous drought duration; the y-axis shows predicted germination probability. The red lines represent the logistic regression lines, and the gray shaded areas are binomial confidence intervals from the logistic regression model.

The combined effects of winter warming and drought stress were assessed in Figure 6, revealing the most severe impacts on *A. deliciosa*. Despite prolonged drought being able to slightly increase the germination probability of *A. deliciosa*, the species’ germination probability still dropped to below 25% under concurrent warming and drought conditions (*p* < 0.001), highlighting its extreme sensitivity to compounded climate stressors. In contrast, *A. rufa* exhibited minimal changes in germination, reinforcing its ecological adaptability and potential as a climate-resilient species. The pronounced vulnerability of *A. deliciosa* suggests that climate change mitigation strategies for kiwifruit cultivation must prioritize adaptive management approaches to maintain germination success.

### 3.7. Validation of Model Predictions

To evaluate the reliability of the classifier models, predicted germination responses were compared with long-term field distribution data for *Actinidia* species in Taiwan [31,32]. The observed distribution patterns aligned strongly with model projections, confirming the robustness of the approach. *A. rufa*, identified as the most resilient species in experimental conditions, was also found to have the broadest natural distribution range, spanning elevations from 50 to 2200 m. Similarly, the high sensitivity of *A. deliciosa* to warming and drought stress was reflected in its restricted high-altitude distribution, indicating that future climate warming may significantly reduce its viable habitat. *A. latifolia*’s latitude-dependent germination responses were also validated by field observations, with southern populations exhibiting lower recruitment success compared to their northern counterparts. The strong correlation between model predictions and real-world distributions underscores the potential application of classifier modeling in forecasting climate change impacts on plant species. These findings provide crucial insights for conservation strategies, breeding programs, and climate adaptation measures for kiwifruit species under future climate scenarios.

## 4. Discussion

This study provides critical insights into the differential seed germination responses of four *Actinidia* species under climate-induced stressors, highlighting the intricate interplay between environmental conditions and the mechanisms underlying seed dormancy. By integrating empirical germination data with classifier modeling, we have demonstrated that species exhibit distinct resilience patterns to warming and drought, underscoring the importance of considering species-specific physiological traits in climate impact assessments.

Among the studied species, *A. rufa* exhibited the highest resilience to climate change stressors, maintaining stable germination percentage under both warming and drought conditions. This suggests that *A. rufa* possesses an ecological dormancy mechanism [33] that enables germination once favorable conditions return, making it an ideal candidate for conservation and ecological restoration efforts. Its widespread natural distribution across various elevations further supports its adaptability, reinforcing the species’ potential to persist under future climate scenarios.

In contrast, *A. deliciosa* emerged as the most climate-sensitive species, with germination percentage plummeting under warming conditions. The sharp decline in germination success under reduced chilling exposure indicates a strong dependency on cold stratification for dormancy release [34,35,36,37,38]. Given the projected rise in winter temperatures, the primary breeding goal for *A. deliciosa* should prioritize the development of cultivars with reduced chilling requirements, rather than improving cold tolerance. Selecting and breeding genotypes that require less chilling for dormancy release would enhance the species’ adaptability to a warming climate and ensure the sustainability of kiwifruit production in regions experiencing reduced winter chill accumulation.

*A. latifolia* demonstrated a latitude-dependent germination response, with populations south of Tropic of Cancer (23.5° N) exhibiting a pronounced decline in germination success under warming conditions. This finding suggests that populations from higher latitudes possess greater thermal resilience, potentially due to localized adaptations to cooler environments [39]. Furthermore, *A. latifolia* exhibited high sensitivity to drought, with extended dry periods leading to significant reductions in germination. The correlation between population distribution and moisture availability aligns with previous ecological observations, emphasizing the necessity of maintaining adequate habitat humidity to ensure species persistence.

*A. setosa* presented the most complex dormancy behavior, with seed germination responses strongly influenced by altitude. Notably, *A. setosa* germination was significantly constrained by seed collection altitude, with higher-elevation populations exhibiting greater germination success. However, despite the variation in germination percentage, cold stratification did not effectively break dormancy in this species, suggesting that its dormancy mechanisms may be more physiologically complex than previously assumed. This altitudinal pattern [40] highlights the potential role of genetic and environmental interactions in dormancy regulation, and the implications for population survival under future climate scenarios should be carefully considered. As climate change accelerates warming trends, populations at lower elevations may experience increased germination failure, necessitating conservation interventions to safeguard genetic diversity.

The predictive models developed in this study were validated against long-term field observations of *Actinidia* species distributions in Taiwan [31,32], demonstrating strong alignment between empirical data and model projections. Traditional species distribution models (SDMs) often fail to incorporate physiological processes [20] such as dormancy-breaking requirements, potentially leading to overgeneralized predictions [17]. Our study highlights the necessity of integrating physiological and ecological data into predictive frameworks to enhance the accuracy of climate impact assessments. The robust model validation underscores its applicability for forecasting species distribution shifts and informing conservation strategies under future climate scenarios.

Beyond its implications for *Actinidia* conservation, this study provides broader insights into the effects of climate change on seed germination dynamics. The findings underscore the need for species-specific conservation planning, as responses to warming and drought are highly variable among species. *A. rufa* emerges as a strong candidate for habitat restoration and assisted migration initiatives, while *A. deliciosa* requires targeted breeding programs to develop low-chilling-requirement cultivars. Additionally, the identification of latitude- and altitude-specific vulnerabilities in *A. latifolia* and *A. setosa* offers valuable guidance for refining conservation priorities and developing adaptive management strategies.

Future research should explore the long-term impacts of climate change on *Actinidia* seedling survival and establishment. While germination success is a critical factor, seedling survival and growth under climate-induced stressors may determine overall population persistence. In particular, the phenomenon of ‘living dead trees’ [2], where mature individuals persist but seedling recruitment fails due to climate stress, warrants further investigation. Such studies should integrate research on bud dormancy, flowering phenology, and other life-stage transitions to formulate comprehensive conservation and agricultural strategies. Collaborating with plant phenology gardens and phenology networks would provide a robust framework for long-term monitoring and enhance the efficiency of climate adaptation research [1], ensuring that mitigation strategies are based on the most comprehensive data available.

In conclusion, our study provides a detailed assessment of *Actinidia* seed germination responses to climate change, offering novel empirical data and predictive modeling insights. The findings contribute to the broader field of plant ecophysiology and climate adaptation, emphasizing the necessity of integrating physiological traits into climate impact assessments. As global temperatures continue to rise, the insights gained from this study will inform conservation efforts, agricultural sustainability, and future research on plant-climate interactions.

## 5. Conclusions

This study provides a clear and concise evaluation of seed germination responses among four *Actinidia* species under climate-induced stressors, demonstrating significant interspecific variation in resilience to warming and drought. Our findings reveal that *A. rufa* exhibits robust ecological adaptability, making it an excellent candidate for ecological restoration under changing climates. Conversely, *A. deliciosa* is notably vulnerable due to its strong chilling requirements, underscoring the urgent need to develop low-chill cultivars to ensure sustainable kiwifruit production.

Additionally, *A. latifolia* shows latitude-dependent vulnerabilities, highlighting the importance of preserving genetic diversity across latitudinal gradients to bolster species resilience. The complex dormancy patterns observed in *A. setosa*, particularly its elevation-dependent germination response, require further investigation into its physiological dormancy mechanisms. Our classifier modeling, validated with long-term field data, effectively predicts species-specific germination outcomes, offering a reliable tool for forecasting the impacts of climate change on plant reproductive success.

Future studies should extend this research by examining seedling establishment and survival, which are crucial for long-term species persistence. Integrating phenological data and physiological traits into climate adaptation strategies will enhance the effectiveness of conservation efforts. Overall, this study provides critical insights into seed germination dynamics under climate change, guiding targeted breeding, conservation, and adaptive management practices for kiwifruit species.

## Figures and Tables

**Table 1:** Information of the 4 species and maximum seed germination percentages.

Species	Collection Sites, Elevations, and GPS	Maximum Seed Germination (%)	Maximum Seed Germination After Cold Stratification (%)
*Actinidia latifolia*	Mutan, Pingtung County elevation 400 m, (22.18262, 120.85061)	43.0	51.5
	Wufeng, Taichung County elevation 200 m, (24.07752, 120.72262)	96.3	94.3
	Peitungyenshan, Nantou County elevation 1600 m, (24.07688, 121.13007)	94.8	94.6
*Actinidia setosa*	Tsuifeng, Nantou County elevation 2250 m, (24.10683, 121.19871)	10.7	14.0
	Siyuan-Yakou, Yilan County elevation 1870 m, (24.39488, 121.35662)	8	16
*Actinidia rufa*	Qingjing, Nantou County elevation 1800 m, (24.06300, 121.16810)	89.3	90.7
*Actinidia deliciosa*	Shengguang, Heping Dist., Taichung City elevation 2500 m, (24.36882, 121.33922)	3.3	93.3

## Data Availability

The datasets generated and analyzed during the current study are not publicly available at this time because they are being used for further ongoing research to develop additional classifier models. Requests to access the datasets should be directed to the corresponding author Ching-Te Chien.
